# Supplementary material for: Integrated Proteomics and Metabolomics Link Acne to the Action Mechanisms of Cryptotanshinone Intervention
Source: Front Pharmacol. 2021 Sep 1;12:700696. doi: 10.3389/fphar.2021.700696 (PMC8440807; doi:10.3389/fphar.2021.700696)
Supplement: Supplementary file 1 [file DataSheet1.ZIP › Supplementary table 1-10 and material 1-3/Material 3-Metlib-MSMS/NEG-Metlib-MSMS/Metlib-MSMS/M185T474_forward/0.994,DL-2-Phosphoglycerate,(M-H)-.pdf]

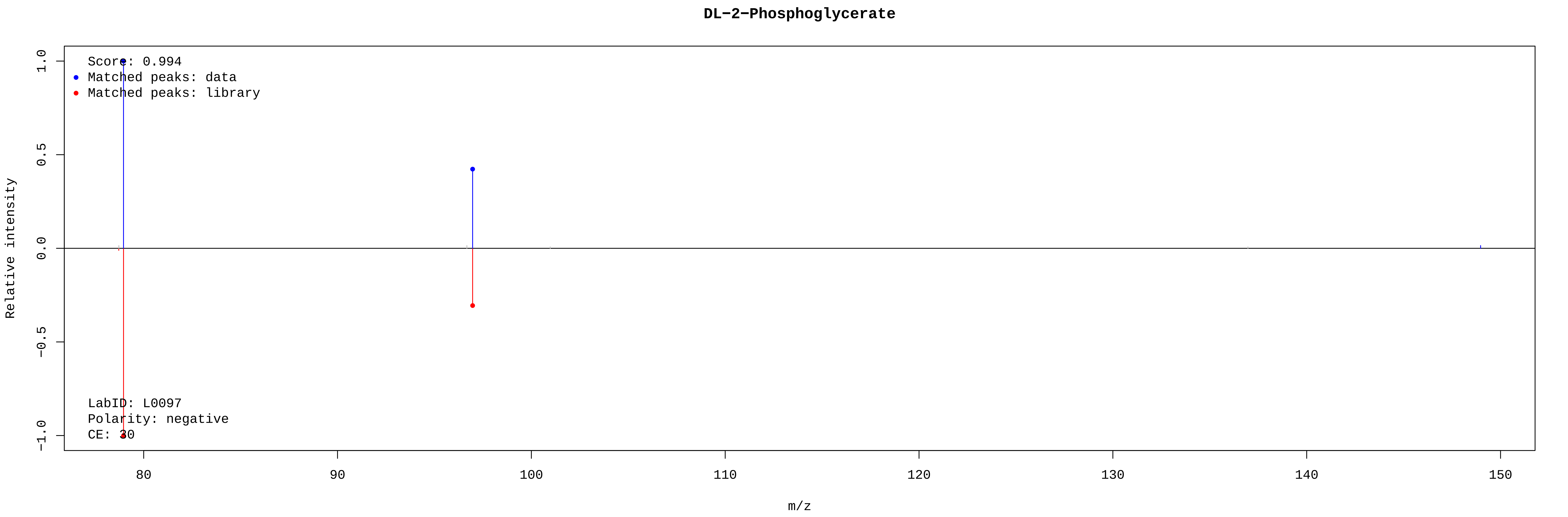
DL−2−Phosphoglycerate
Score: 0.994
Matched peaks: data
Matched peaks: library
LabID: L0097
Polarity: negative
CE: 20
Relative intensity
1.0
0.5
0.0
−0.5
−1.0
80
90
100
110
120
130
140
150
m/z
